# Half-Yearly Report of Cases in Midwifery, Which Have Occurred in the Northern District of the London and Southwark Midwifery Institution

**Published:** 1829-02

**Authors:** C. Waller

**Affiliations:** Consulting Accoucheur to the above Institution, and Lecturer on Midwifery at the Medical School, 58, Aldersgate street.


### MIDWIFERY.

*Half-yearly Report of Cases in Midwifery, which have occurred in the Northern District of the London and Southwark Midwifery Institution.* By C. Waller, Esq. Consulting Accoucheur to the above Institution, and Lecturer on Midwifery at the Medical School, 58, Aldersgate street.

The following is a correct list of the returns which have been made to me within the last six months. Some allowance must, of course, be made for those letters which either have not been returned, on the one hand, or have been mislaid during the half year, on the other.

| Number of Women delivered. | Sex of Children. Males. | Females. | Born alive. | Stillborn. | Presentation. |
|---|---|---|---|---|---|
| | | July, 1828. | | | |
| 27 | 11 | 16 | 27 | 0 | Natural |
| | | August. | | | |
| 22 | 13 (1 twin case) | 10 | 23 | 0 | 22 Natural<br>1 Breech |
| | | September. | | | |
| 24 | 11 (1 twin case) | 14 | 25 | 0 | 22 Natural<br>2 Breech<br>1 Face |
| | | October. | | | |
| 25 | 15 | 10 | 24 | 1 | 24 Natural<br>1 Footling |
| | | November. | | | |
| 30 | 22 | 8 | 30 | 0 | 29 Natural<br>1 Breech |
| | | December.* | | | |
| 3[illegible] | 14 | 19 | 31 | 2 | 32 Natural<br>1 Breech |
| Total, 160 | 86 | 77 | 160 | 3 | |

Besides these, several cases of abortion have occurred in the institution within these few months; in one or two instances, attended with large losses of blood; the patients, however, eventually recovering very well.

*Remarks.*—Several cases of interest have occurred since my last report. I shall first relate those which were attended with hemorrhage.

Case I.—Mrs. H., æt. twenty-five, when taken in labour, was seized with rather a profuse hemorrhage. I was requested, by the pupil in attendance, to visit her, and found her extremely low and faint, countenance pale, pulse very feeble. In consequence of the repeated application of cold, the bleeding had nearly ceased. On examination, I found the os uteri but little dilated, the vertex presenting, no placenta to be felt, and the membranes unbroken; the patient had slight labour pains. As a measure of precaution, I ruptured the membranes, and there was no return of the discharge, the patient being safely delivered, in a few hours afterwards, of a living child.

Case II.—Mrs. J. had been delivered about two hours: the placenta not coming away, my assistance was requested. A little before my arrival at the house, a very profuse hemorrhage came on. On applying cold and friction, the uterus was made to con-

* One twin case, in which the patient was delivered of a male and female child.

tract; but the placenta not being expelled, I introduced my hand into the uterus for the purpose of removing it, and found it pretty firmly adherent throughout the greater part of its extent. The hemorrhage immediately ceased on its removal; but the patient remained faint, and covered with a clammy perspiration for several hours. The pulse did not rise much in frequency during the bleeding, but reaction followed, and it continued rapid for several days. Besides this, there was no unpleasant symptom.

Case III.—Was requested to see Mrs. R., who had been delivered about half an hour. The birth of the child was followed by a very sudden and profuse hemorrhage. I found her with a very ghastly countenance, and with a pulse scarcely perceptible. A little stimulus was given, and friction employed, which caused the womb to contract to a certain extent, though not sufficiently as to expel the placenta. I consequently introduced the hand to remove it, when the bleeding immediately ceased.

In this case there was no hemorrhagic reaction, the pulse, after she rallied, remaining steadily at eighty. For some time, however, this patient complained of extreme chilliness.

Case IV.—Mrs. J. was delivered of twins, and, before the expulsion of the placenta, had lost a very large quantity of blood; which, however, appeared to have no effect upon the general constitution. The uterus appeared to contract well, and the placenta readily came away, every thing appearing to be going on well. In about ten minutes, however, she was seized with a deadly syncope, although no blood passed out of the vagina. On examining the uterus through the abdominal covering, it was found to be slightly enlarged, though to no great extent. On pressing firmly upon the fundus uteri, so as to double it, as it were, upon itself, a coagulum of blood, of about eight ounces, was expelled: there was no relaxation afterwards, and the patient did well, though she remained faint for some hours.

This case is interesting, as showing that, after a patient has lost a certain quantity of blood, how small a proportion in addition may produce dangerous symptoms; and is therefore a negative argument in favor of the transfusion of a quantity of blood, small in comparison to what has been lost, in order that the scale may be turned in the patient's favor.

Several other slighter cases of hemorrhage have occurred, in all of which the application of cold, combined with external friction, was sufficient.

Two or three cases of peritoneal inflammation have also happened, which were readily cured by the free use of the lancet, and the employment of calomel and opium.

In one case the child had a large tumor on the scalp, which burst before birth, and a large quantity of grumous blood was discharged. I was requested to visit this case, the pupil conceiving, from the quantity of blood which passed away, that it was a presentation of the placenta. The precise nature of the case was not quite obvious till after the child was born, when it was observed that the tumor had occupied a considerable portion of the summit of the cranium, as the torn scalp was there greatly overlapping. Pressure was applied, and, after many hours, the hemorrhage from the wound was restrained; but the child died on the fourth day. It is a curious fact, that there was a very considerable quantity of hair growing from every part of the scalp except on the site of the tumor, and *there* it was perfectly bald.

A case of fatal inflammation, following a very severe labour, has occurred. The notes of this case were very correctly taken by my friend and pupil Mr. Harris Dunsford, and I therefore subjoin it in his own words.

"Mrs. G., aged thirty-two, of pale unhealthy appearance, a patient of the London and Southwark Institution, sent for me to attend her on Wednesday, November the 19th, at six in the evening. She has had two children before, both of which were stillborn, and instruments were employed. The membranes burst the evening before I saw her, at eight o'clock, but the pains remained very trifling, occurring at long intervals. The os uteri was dilated to the size of a sixpenny piece, hard and thin. The next day (Thursday), at ten o'clock, the pains became much more severe, and came on every ten minutes or quarter of an hour, but they were confined to the loins, front and inside of the thighs. The mouth of the womb was dilated to the size of a halfcrown, and the funis presented, pulsating strongly. It was observed that the funis, a foot, a hand, and the head presented. The promontory of the sacrum was felt projecting considerably towards the pubis. The woman was very desponding, and frequently said she should never recover.

"Messrs. Waller and Doubleday saw her in the evening, (Thursday,) and were of opinion that she could not possibly be delivered by the unassisted efforts of nature, the pelvis being distorted in a high degree. Accordingly, Mr. Waller immediately proceeded to empty the uterus: pushing back the hand, he took hold of the foot, and after some difficulty brought down the leg: but, although considerable efforts were used, the breech could not be made to pass. At length, the cord no longer pulsating, it was agreed to perforate the head, which was soon effected. Extension was now again employed on the leg, but without effect. The head was perforated in another part, and a piece of calico was tied round the leg, to assist in drawing down the breech; which at length passed the upper aperture.

"The patient's pulse hitherto had been soft, slow, and regular; but after this, and during the passage of the head, which did not come through without considerable difficulty, it increased greatly in frequency, and the woman now became very restless. The uterus did not contract immediately. A dose of the secale cornutum in powder, and a little gin-and-water, were given, and it then contracted pretty well, and the placenta was expelled. Two hours afterwards, forty drops of laudanum were given her.

"She did not sleep, but during the night became extremely restless, and had frequent attacks of faintness. I was fetched at four A.M., and found her with a pale, anxious countenance, hot and dry skin; furred tongue, moist at the edges, but dry in the centre; quick and feeble pulse, beating 120 in the minute. She complained of *a violent pain at the pit of the stomach*, and of soreness over the whole abdomen, increased on pressure. She has vomited once, and complains of slight nausea; is much annoyed by wind. Mr. Doubleday saw her soon after, and ordered twelve leeches to be applied to the pit of the stomach, and the following pills, one to be taken every second hour:

℞. Opii gr. iij.; Hydr. Submur. gr. vj.; Antim. Tart. gr. iss. M. fiant pil. iij.

"Saw her again at twelve, and at four P.M. The leeches produced a little relief, but the pain still continued very severe; the pulse sometimes intermits; the mouth is parched, but she is unwilling to drink, as she says it increases the pain; the tongue is dry and brown, and she is constantly smacking her lips. She talks sometimes incoherently; her countenance is expressive of anxiety, and the skin covered with perspiration. The abdomen is much swollen, and there appears to be fluid in the cavity. She makes water without difficulty; the lochia have entirely ceased. Strong beef tea is given from time to time.

"Mr. Waller saw her this evening (Friday), at ten: the pulse was at 112, feeble, but regular; the countenance more natural. He ordered the following pills and mixture:

℞. Opii gr. i.; Hydr. Submur. gr. ij. M. fiat pil. secunda quaque hora sumend. cum cochleariis iij. majoribus mist. sequent.

℞. Sodæ Tartarizatæ ℥iss.; Syrup. Papav. ℥ss.; Aquæ Menth. Pip. ℥viij. M. fiat mist.

"Nov. 22d, eight A.M. (Saturday).—The patient has dozed a little during the night. Has occasional twitchings of the muscles of the balls of the eyes; the tongue is parched, and rather brown; pulse 108, regular, and more firm. Still complains of the pain in the epigastrium and abdomen, which is very much distended and tympanitic. The skin is hot and dry; she brings up every thing she takes. A turpentine injection was given, and brought off some wind.

"In the evening, the pain in the epigastrium had ceased. A common injection was then administered, and feces were discharged.

"Mr. Waller saw her at night: the pulse was quicker and more feeble, and the countenance had assumed a cadaverous appearance.

"About an hour and a half before her death, which took place on Sunday, at one A.M., the pain in the abdomen (as the attendants state) ceased suddenly."

*Sectio cadaveris*, ten hours after death.—The abdominal parietes were excessively distended, but as yet putrefaction had not begun to take place. On opening the cavity, the whole of both of the peritoneal surfaces were observed to be highly inflamed, and extensively, nay almost generally, adherent to each other. A considerable clot of blood was resting on the omentum, which was tightly adherent to the intestines, among which was a large quantity of bloody serum. The uterus was large and flabby, and distended with air; its internal surface presented a uniform appearance of bloodshot vascularity, (perhaps the stain of the lochia contributed to this effect,) which towards the cervix was very dark; and here there was an uneven and somewhat lobulated appearance. (Was this owing to disease of the glands?) The vagina appeared to participate in the inflammation; as did also the bladder and rectum.

Perhaps it may be asked, why more active depletion was not in this case had recourse to? To this I would reply, that, so far as my experience in these cases goes, the disease itself, when of an extensive nature, is attended with such a complete prostration of the patient's strength, marked by the rapidity and perfectly powerless state of the pulse, that venesection appears to hasten the fatal event. I have seen several cases tending to prove this point.

The face presentation happened to a young woman in labour with her first child, and consequently the process of parturition was considerably protracted, and her sufferings increased; but the child was born living, and the mother had a good getting up.

In several cases, (some in the Institution, and some in my private practice,) I have administered the secale cornutum, and its effect continues to justify the favorable opinion I have before given of its powers. I find that the powder in doses of half a drachm, repeated (if necessary) in a quarter of an hour, is usually sufficient to answer the purpose.

In one instance, obstinate diarrhœa succeeded an attack of fever, which had resisted every means of cure; and the poor woman is now (seven weeks after her delivery) gradually sinking. The sulphate of copper, with opium, was used among other remedies, but without success.*

* A few days after the above was written the patient died; and, on examination, very extensive disease of the mucous glands of the large intestines was found to have taken place: some of them were as large as a pea. A considerable quantity of feculent matter was locked up in the colon.

In one instance there was repeated hemorrhage from the umbilical cord, which so weakened the infant that it died in a few days. This, I apprehend, must have arisen from disease of the arteries, as there were no less than six ligatures applied, and by three different persons, (myself among the rest;) notwithstanding which, however, the bleeding recurred at intervals, and the blood was observed to issue not from the part at which the ligature was applied, but from the extremity of the funis.

---

